# Cathepsins L and B target HIF1α for oxygen-independent proteolytic cleavage

**DOI:** 10.1038/s41598-024-65537-9

**Published:** 2024-06-26

**Authors:** Sarah Stuart, Daniel Tarade, Michael Ohh

**Affiliations:** 1 Department of Laboratory Medicine & Pathobiology, University of Toronto, 1 King’s College Circle, Toronto, ON M5S 1A8 Canada; 2 Department of Biochemistry, University of Toronto, 661 University Avenue, Toronto, ON M5G 1M1 Canada

**Keywords:** Biochemistry, Molecular biology

## Abstract

The oxygen-labile transcription factor called hypoxia-inducible factor (HIF) is responsible for the cellular and organismal adaptive response to reduced oxygen availability. Deregulation of HIF is associated with the pathogenesis of major human diseases including cardiovascular disease and cancer. Under normoxia, the HIFα subunit is hydroxylated on conserved proline residues within the oxygen-dependent degradation domain (ODD) that labels HIFα for proteasome-mediated degradation. Despite similar oxygen-dependent degradation machinery acting on HIF1α and HIF2α, these two paralogs have been shown to exhibit unique kinetics under hypoxia, which suggests that other regulatory processes may be at play. Here, we characterize the protease activity found in rabbit reticulocytes that specifically cleaves the ODD of HIF1α but not HIF2α. Notably, the cleavage product is observed irrespective of the oxygen-dependent prolyl-hydroxylation potential of HIF1α, suggesting independence from oxygen. HIF1α M561T substitution, which mimics an evolutionary substitution that occurred during the duplication and divergence of HIF1α and HIF2α, diminished the cleavage of HIF1α. Protease inhibitor screening suggests that cysteine proteases cathepsins L and B preferentially cleave HIF1αODD, thereby revealing an additional layer of differential HIF regulation.

## Introduction

The hypoxia-inducible factor (HIF) family of transcription factors are responsible for the cellular and organismal response to hypoxia. In mammals, the three paralogs of the HIFα subunit can each dimerize with the constitutively expressed HIFβ subunit to form a functional HIF transcription factor. Despite HIF1 and HIF2 both binding identical hypoxia-responsive elements (HRE; 5’-RCGTG-3’), they are not redundant^1,2^. HIF1 appears to play a key role in the acute metabolic response to hypoxia by increasing glycolytic flux and decreasing mitochondrial respiration, while HIF2 positively regulates the hormone erythropoietin, which signals erythropoiesis, and stem cell factors like Oct4^3^.

All HIFα paralogs possess an oxygen-dependent degradation domain (ODD). The two conserved proline residues within this disordered region are targets of the prolyl-hydroxylase domain-containing enzyme (PHD) family. In the presence of sufficient oxygen, PHDs catalyze the hydroxylation of these proline residues^4,5^. The hydroxyproline moieties are then recognized by von Hippel-Lindau protein (pVHL), the substrate-conferring component of an E3 ubiquitin ligase complex^6,7^. Thus, molecular oxygen is linked to the protein stability of HIFα, whose half-life is approximately 5 min under normoxia^8^. Notably, the deregulation of HIF has been associated with the pathogenesis of major human diseases such as cardiovascular disease, stroke and cancer, and numerous mutations in genes encoding the key components of the oxygen-sensing pathway such as *EGLN1*, *EPAS1* and *VHL* have been shown to cause Pacak-Zhuang syndrome and VHL disease, characterized by the development of neuroendocrine tumours, haemangioblastoma and polycythemia^3^.

Despite HIF1α and HIF2α both being regulated by PHDs and pVHL in a similar manner, they have been reported to display a different activation profile under hypoxia, where HIF1α protein level generally peaks rapidly and declines via an unclear mechanism following 24 h under hypoxic conditions while HIF2α remains chronically activated^9–11^. There has been previous research into this hypoxic degradation of HIF1α. One model focuses on a negative feedback loop, whereby mitochondrial respiration decreases and PHD activity increases such that HIFα degradation outpaces synthesis due to increased oxygen availability^10,12–16^. Conversely, several groups have attributed the hypoxic degradation to the activity of other negative regulators^17–27^. Notably, VHL-dependent and -independent degradation under hypoxic conditions is not necessarily mutually exclusive. Indeed, HIF1α is degraded under hypoxic conditions even in *VHL*-null cells via unclear mechanisms^27^.

We recently reported on a biochemical and structural study that explored the effect of evolutionarily relevant ODD domain substitutions in HIF1α and HIF2α. In particular, we focused on the amino acid three residues N-terminal to the primary hydroxylation site (P564 and P531 in HIF1α and HIF2α, respectively)^28^. HIF1α Met_n-3_ and HIF2α Thr_n-3_ are conserved among species expressing both proteins. One biochemical phenotype associated with this substitution is a two-fold decrease in HIF2α affinity for pVHL compared to HIF1α. We have previously shown via co-crystallization of HIF2αODD peptide with pVHL that HIF1α Met_n-3_ stabilizes the interaction more than HIF2α Thr_n-3_ due to solvation effects^28,29^.

Despite a clear structural basis and strong evolutionary signature, it remains unclear if this divergence between HIF1α and HIF2α is biologically significant. We speculated that the increased affinity of pVHL for HIF1α might explain the preferential degradation of HIF1α under chronic hypoxic conditions, where oxygen demand decreases due to mitophagy and increased available O_2_ levels allow for increased hydroxylation. In addition to its unambiguous impact on pVHL affinity, we observed a previously unappreciated cleavage of HIF1α containing Met_n-3_ while HIF2α with Thr_n-3_ appeared to escape proteolytic cleavage via unknown proteases present in rabbit reticulocytes^28^. Given the strong selection that maintains HIF1α Met_n-3_ and HIF2α Thr_n-3_ among vertebrate species, we rationalized that this phenotype may affect the regulation of HIF transcription factors.

Here, we characterize a protease activity found in rabbit reticulocyte lysate toward recombinant HIF1αODD that is dependent on Met_n-3_. Cleavage was not significantly influenced by the hydroxylation potential of the conserved proline residues within the ODD, suggesting independence from oxygen, and a systematic screen of protease inhibitors identified members of the cathepsin family of cysteine proteases to play a role, at least in part, in the preferential cleavage of HIF1αODD.

## Results

### HIF1α, but not HIF2α, ODD is cleaved by protease activity in rabbit reticulocytes

**Figure 1:**
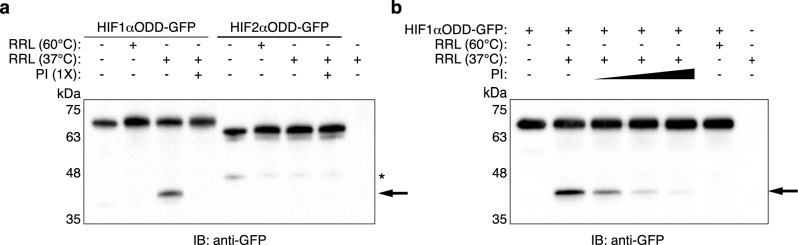
HIF1α, but not HIF2α, ODD is cleaved by rabbit reticulocyte protease. (**a**) Rabbit reticulocyte lysate (RRL) was pre-incubated for 5 min at either 37 °C or 60 °C, with or without protease inhibitor cocktail (PI), as indicated. Reactions were then incubated with recombinant HIF1αODD-GFP or HIF2αODD-GFP for 60 min at 37 °C, resolved by SDS-PAGE, and immunoblotted for GFP. (**b**) Recombinant HIF1αODD-GFP was incubated for 60 min at 37 °C with rabbit reticulocyte lysate that had been pre-incubated for 5 min at 37°C or 60°C, in the absence or presence of protease inhibitor cocktail, as indicated. PI was added at 0.1X, 0.5X or 1X the manufacturer’s recommended usage. Reactions were resolved on SDS-PAGE and immunoblotted for GFP. Arrow indicates cleaved HIF1αODD-GFP. Asterisks indicate non-specific protein bands. Blots are representative of three independent experiments. Original blots are presented in Supplementary Fig. S2.

We previously observed that when HIF1αODD was expressed in an in vitro transcription/translation (IVTT) system, the resulting protein migrated as two distinct bands on SDS-PAGE^28^. This observation led us to hypothesize that the faster-migrating species represented a cleavage product of the HIF1αODD. To characterize this potential cleavage, we purified HIF1αODD-GFP and HIF2αODD-GFP. Incubation of recombinant HIF1αODD-GFP with rabbit reticulocyte lysate resulted in the appearance of a faster-migrating fragment that markedly diminished when co-incubated with a protease inhibitor cocktail or when rabbit reticulocyte lysate was heat-inactivated at 60°C (Fig. 1a). In contrast, HIF2αODD did not appear to be cleaved when incubated with rabbit reticulocyte lysate, regardless of a temperature setting of 37 °C or 60 °C (Fig. 1a). Increasing concentrations of protease inhibitor cocktail led to corresponding decreases in the amount of cleaved HIF1αODD-GFP (Fig. 1b). The faster-migrating species was specific to HIFαODD-GFP as these cleaved products were not detected when rabbit reticulocyte lysate was incubated in the absence of purified HIFαODD-GFP protein (Fig. 1a,b). These results confirm our earlier in vitro transcription/translation observations and suggest that the lower HIF1αODD-GFP protein product is the result of proteolytic activity contained in rabbit reticulocyte lysate.

### Cleavage of HIF1αODD is dependent on M561 irrespective of prolyl-hydroxylation status

**Figure 2:**
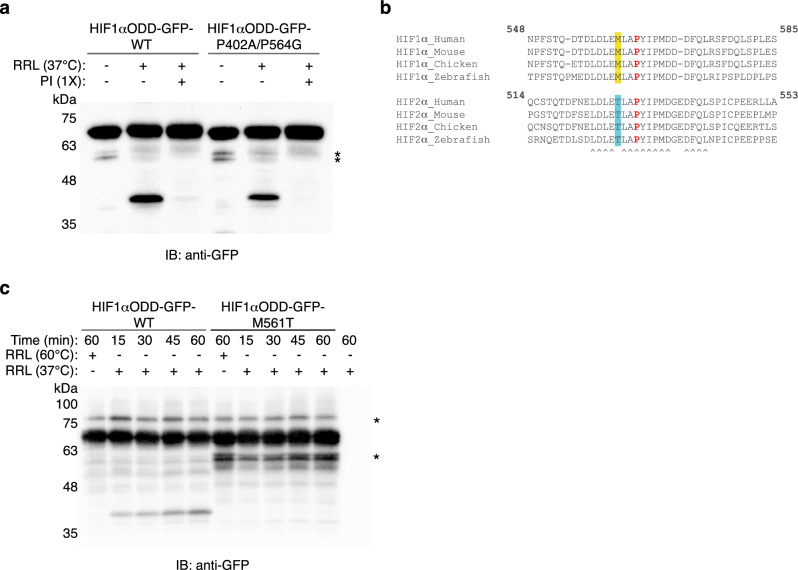
Cleavage of HIF1α ODD is dependent on HIF1α M561 in an oxygen-independent manner. (**a**) Rabbit reticulocyte lysate (RRL) was pre-incubated for 5 min at 37 °C, with or without protease inhibitor cocktail (PI), as indicated. Reactions were then incubated with recombinant HIF1αODD-GFP-WT or HIF1αODD-GFP-P402A/P564G for 60 min at 37 °C, resolved by SDS-PAGE, and immunoblotted for GFP. (**b**) Alignment of HIF1α and HIF2α residues surrounding the primary hydroxylation site (shown in red). ^ indicates conserved residues; Met_n-3_ in HIF1α is highlighted in yellow and Thr_n-3_ in HIF2α is highlighted in blue. (**c**) Recombinant HIF1αODD-GFP-WT or HIF1αODD-GFP-M561T was incubated at 37 °C with rabbit reticulocyte lysate for the indicated times. Reactions were resolved by SDS-PAGE and immunoblotted for GFP. Asterisks indicate non-specific protein bands. Blots are representative of three independent experiments. Original blots are presented in Supplementary Fig. S2.

We next asked whether the oxygen-dependent hydroxylation of HIF1αODD was necessary for its cleavage via the protease activity in rabbit reticulocytes. Mutation of the two hydroxylation sites, P402 and P564, did not markedly prevent the appearance of cleaved product, suggesting that prolyl-hydroxylation was not required (Fig. 2a). Based on the differential cleavage pattern between the two paralogs, we focused on HIF1α M561; this amino acid in HIF1α is evolutionarily conserved among vertebrate species (Fig. 2b), and the corresponding substitution to a conserved T528 in HIF2α results in decreased affinity to pVHL^28^. Notably, HIF1αODD(WT)-GFP cleavage occurred within 15 min while no cleavage of the M561T substitution mutant was detected over the course of the 1 h experiment (Fig. 2c). These results suggest that the HIFα paralogs are differentially cleaved, and that this cleavage is, at a minimum, dependent on the M561 residue.

### HIF1αODD is cleaved by rabbit reticulocyte cysteine protease

**Figure 3:**
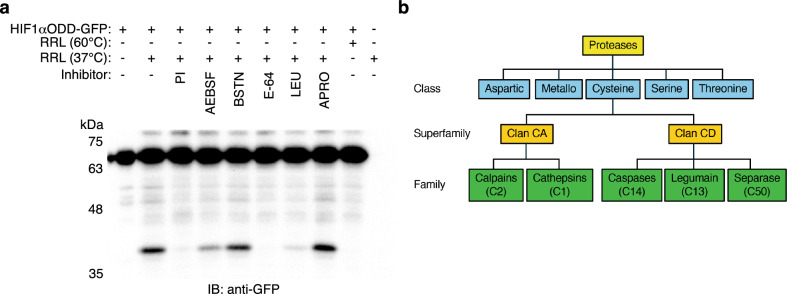
HIF1αODD is cleaved by rabbit reticulocyte cysteine protease. (**a**) Rabbit reticulocyte lysate (RRL) was pre-incubated for 5 min at 37 °C or 60 °C, then incubated for 60 min at 37 °C with HIF1αODD-GFP and 1X protease inhibitor cocktail (PI), 2 mM AEBSF, 130 μM bestatin (BSTN), 14 μM E-64, 1 μM leupeptin (LEU) or 0.3 μM aprotinin (APRO), as indicated. Reactions were resolved by SDS-PAGE and immunoblotted for GFP. Blot is representative of three independent experiments. Original blot is presented in Supplementary Fig. S2. (**b**) Summary of MEROPS system of protease classification^30^. Proteases are first grouped into classes (aspartic, metallo, cysteine, serine or threonine) based on a common catalytic mechanism. Classes are divided into superfamilies, or clans, based on a common evolutionary origin; MEROPS contains 14 cysteine protease clans, with nearly all mammalian cysteine proteases belonging to either Clan CA or Clan CD. Clans are further grouped into families based on amino acid sequence similarity. Clan CA contains the papain-like enzymes, including calpain (C2) and cathepsin (C1) families, while Clan CD is notable for containing the caspase (C14) family, as well as mammalian legumain (C13) and separase (C50).

To determine the protease responsible for cleavage, we incubated HIF1αODD-GFP with rabbit reticulocyte lysate in the absence or presence of the individual components of the protease inhibitor cocktail. Metalloprotease inhibitor bestatin and serine protease inhibitor aprotinin had negligible effect, while serine protease inhibitor AEBSF showed only modest effect on reducing the level of the faster-migrating HIF1αODD fragment (Fig. 3a). In contrast, E-64 and leupeptin strongly reduced the level of cleaved HIF1αODD product (Fig. 3a). The rabbit reticulocyte lysate pre-incubated at 37°C, but not at 60°C, generated the expected cleaved HIF1αODD product that was blocked in the presence of protease inhibitor cocktail (Fig. 3a). Leupeptin inhibits serine/threonine and cysteine proteases, while E-64 is specific to cysteine proteases, suggesting that HIF1αODD is cleaved, at a minimum, by a cysteine protease present in the rabbit reticulocyte lysate.

### Cathepsins L and B cleave HIF1αODD

**Figure 4:**
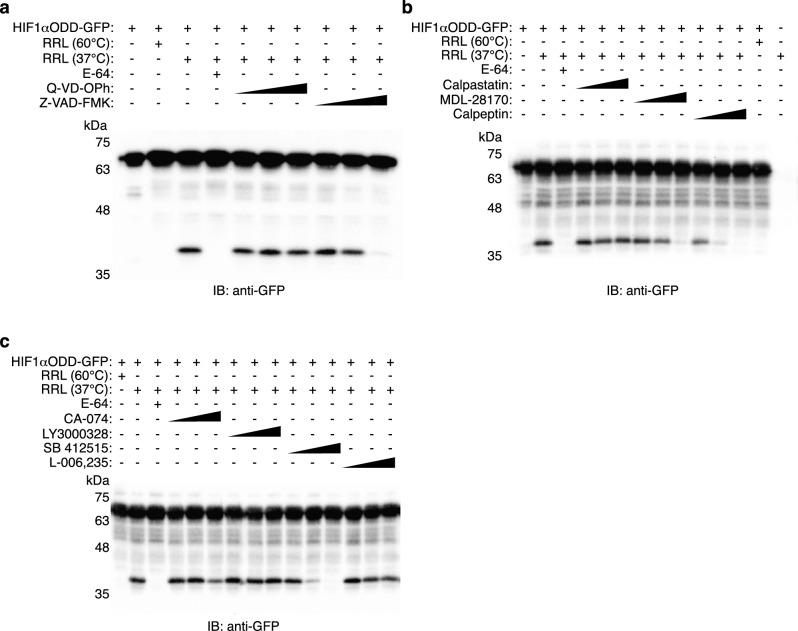
HIF1αODD cleavage is reduced by cathepsins L or B inhibitors. (**a**–**c**). Rabbit reticulocyte lysate (RRL) was pre-incubated for 5 min at either 37 °C or 60 °C, with DMSO or inhibitor, as indicated. Recombinant HIF1αODD-GFP was added and incubated for 60 min at 37 °C. Reactions were resolved by SDS-PAGE and immunoblotted for GFP. E-64 was used at 10 μM; Q-VD-OPh, Z-VAD-FMK, calpastatin peptide, MDL-28170 calpeptin, CA-074 (CTSB inhibitor), LY3000328 (CTSS inhibitor), SB 412515 (CTSL inhibitor), and L-006,235 (CTSK inhibitor) were each used at 0.1, 1, and 10 μM. Blots are representative of three independent experiments. Original blots are presented in Supplementary Fig. S2.

We next attempted to further characterize the cysteine protease responsible for HIF1αODD cleavage. The MEROPS Peptidase Database classifies proteases of similar evolutionary origin into distinct superfamilies or clans^30^. The two major superfamilies of cysteine proteases present in mammals are Clan CA, which includes the cytoplasmic calpain family and the lysosomal cathepsins, and Clan CD, which contains the caspases as its most prominent members^30,31^ (Fig. 3b). We evaluated their involvement with the use of small molecule inhibitors. Incubation with pan-caspase inhibitor Q-VD-OPh did not prevent HIF1αODD-GFP cleavage by rabbit reticulocyte lysate (Fig. 4a), in agreement with previous reports that E-64 inhibits the CA clan but not the CD clan of cysteine proteases^32–34^. We noted a reduction in cleavage using a different pan-caspase inhibitor, Z-VAD-FMK (Fig. 4a); however, this may be due to off-target effects as Z-VAD-FMK is less selective and has been shown to inhibit cathepsins and calpains^35–39^.

We next examined the calpain family using three different inhibitors. Calpastatin is an endogenous inhibitor of calpains^40^; therefore, we utilized acetyl-calpastatin (184–210), a synthetic 27-mer peptide derived from calpastatin exon 1B that strongly inhibits calpains I and II, and is highly selective for calpain over other cysteine proteases^41,42^. In addition, MDL-28170 and calpeptin both inhibit calpain I and calpain II^43,44^; however, MDL-28170 retains activity towards cathepsins B and L^45,46^, while calpeptin potently inhibits cathepsins L, B and K^47–49^. At higher concentrations, both MDL-28170 and calpeptin reduced or prevented HIF1αODD-GFP cleavage while calpastatin, in contrast, had no effect (Fig. 4b). Given the reduced specificity of MDL-28170 and calpeptin compared to calpastatin, we surmised that calpains were unlikely to be responsible for cleavage, leaving the possibility of cathepsins in the cleavage of HIF1αODD.

We next performed the cleavage reaction with a selection of inhibitors directed against members of the cysteine cathepsin family. Given that cathepsin B has previously been implicated in the regulation of HIF1α^17,18^, and both cathepsins B and L have been identified as HIF1α targets^50,51^, we hypothesized that HIF1-mediated cathepsin expression may represent a negative feedback loop. We utilized inhibitors of cathepsin B (CA-074) and cathepsin L (SB 412515) in our panel, as well as inhibitors of cathepsin S (LY3000328) and cathepsin K (L-006,235). Despite the restricted tissue expression of cathepsins K and S^52–55^, hypoxia has been implicated in the regulation of cathepsin K and bone resorption^56–59^, while cathepsin S intriguingly retains activity across a broad pH range^60–63^, similar to the rabbit reticulocyte protease that cleaves HIF1αODD-GFP (Supplementary Fig. S1). Cathepsin S inhibitor LY3000328 and Cathepsin K inhibitor L-006,235 showed negligible reduction in HIF1αODD-GFP cleavage at any concentrations tested (Fig. 4c). In contrast, inhibitors toward the ubiquitously-expressed cathepsins B and L diminished cleavage by rabbit reticulocyte lysate; cathepsin B inhibitor CA-074 reduced cleavage at 10 μM, while cathepsin L inhibitor SB 412515 reduced cleavage even at 0.1 μM (Fig. 4c). These results suggest that cathepsins B and/or L are involved in the cleavage of HIF1αODD.

**Figure 5:**
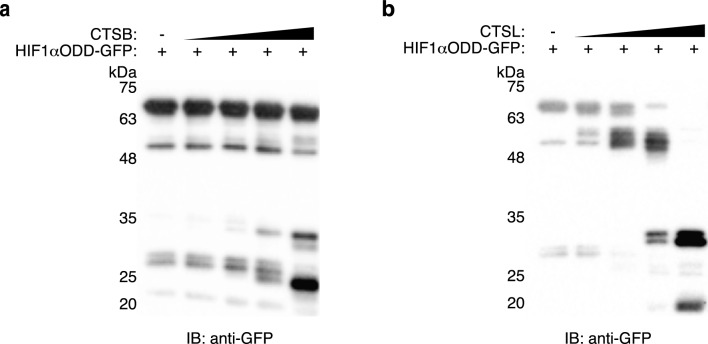
HIF1αODD is cleaved by recombinant human cathepsins L or B. HIF1αODD-GFP-WT was incubated for 1 h at 37 °C in 50 mM MES pH 6 containing 5 mM DTT and increasing concentrations (0.16, 0.8, 4, 20 ng) of recombinant human cathepsin B (**a**) or increasing concentrations (0.016, 0.08, 0.4, 2 ng) of recombinant human cathepsin L (**b**). Reactions were resolved on SDS-PAGE and immunoblotted for GFP. Blots are representative of three independent experiments. Original blots are presented in Supplementary Fig. S2.

We next asked whether recombinant cathepsins B and L could directly target HIF1αODD for proteolytic cleavage. Purified HIF1αODD-GFP incubated at pH 6 with recombinant human cathepsins B or L produced several faster-mobility products with corresponding reduction of the full-length proteins in a dosage-dependent manner (Fig. 5). Although the human cathepsins B and L produced a different cleavage product profile compared to that generated via the enzymatic activity present in the rabbit reticulocytes, these results taken together with the above inhibitor study support the notion that cathepsins B and/or L are involved in the cleavage of HIF1αODD.

## Discussion

The discovery of the PHD-HIFα-pVHL axis revealed a robust mechanism by which changes in intracellular oxygen tension correspondingly regulate the protein stability and thereby the level of HIFα. There are, however, unique differences in the kinetics of HIF1α and HIF2α activity that cannot be readily accounted for by this understanding. For example, HIF1α is generally regarded as an early responder where it regulates the key enzymes of the glycolytic pathway in response to acute metabolic stress, while HIF2α regulates genes such as *EPO* encoding erythropoietin in response to chronic hypoxic stress to increase the level of circulating oxygen-carrying red blood cells. Concordantly, unlike HIF2α, which remains high following its stabilization under hypoxia, HIF1α level has been shown to precipitously decline under hypoxia following its rapid stabilization. This intriguing phenomenon of HIF1α is thought to be pVHL-independent as its decline occurs under hypoxia, a condition that prevents pVHL recognition of HIFα.

Here, we characterize a proteolytic activity found in rabbit reticulocyte that is specific to HIF1α independent of oxygen-mediated prolyl-hydroxylation status. By screening protease inhibitors, we identified members of Clan CA of cysteine proteases that are capable of cleaving HIF1αODD. Notably, the cleavage was dependent on the evolutionarily conserved M561 found only in HIF1α. The M561T substitution mimics the natural divergence between HIF1α and HIF2α, and we have previously reported that this substitution decreases the affinity of HIF1α for pVHL by roughly two-fold^28^. Thus, given the strong evolutionary signature this substitution displays in vertebrate species, the specific cleavage of HIF1α and not HIF2α may fine-tune the oxygen-sensing pathway.

Cathepsin B has previously been implicated in the regulation of HIF1α^17,18^, and both cathepsins B and L have been identified as HIF1α targets^50,51^. We show here that inhibitors to cathepsins B and L can decrease the level of cleaved HIF1αODD product by a protease activity found in rabbit reticulocyte while recombinant human cathepsins B and L can cleave purified HIF1αODD at multiple sites in a dosage-dependent manner. We hypothesize that HIF1-mediated cathepsin expression may represent a natural negative feedback loop that may explain the decrease in HIF1α expression following an early upregulation of HIF1α during hypoxia^9–11,64^. Although further studies will be required to validate and extend the present work *in cellulo* and in vivo, we suggest that cathepsins L and B may play a role in the negative regulation of HIF1α in conditions where oxygen is limited.

## Methods

### Plasmids

pET21b-HIF1αODD-cp8GFP-35-His_6_ and pET21b-HIF2αODD-cp8GFP-35-His_6_ were synthesized by GenScript. Design of the constructs was modified from Martinez-Fonts et al.^65^. Briefly, codon optimized genes encoding for amino acids 387–581 of HIF1α, or amino acids 390–544 of HIF2α were fused to a circular permutant of superfolder GFP with a disordered 35 amino acid tail and a 6X His tag. The synthesized gene products were inserted into pET21b( +) between *Nde*I and *Bam*HI. QuikChange site-directed mutagenesis (Agilent) was used to introduce M561T (primers 5’– CGGATTTGGACCTTGAAACCCTGGCGCCTTACATTCCAATG – 3’ and 5’ – CATTGGAATGTAAGGCGCCAGGGTTTCAAGGTCCAAATCCG – 3’), P402A (primers 5’ – GACATTGCTTGCGGCCGCGGCTGGGGACAC – 3’ and 5’ – GTGTCCCCAGCCGCGGCCGCAAGCAATGTC – 3’) and P564G (primers 5’ – GGACCTTGAAATGCTGGCGGGTTACATTCCAATGGACGAC – 3’ and 5’ – GTCGTCCATTGGAATGTAACCCGCCAGCATTTCAAGGTCC – 3’) mutations into HIF1α.

### Reagents

Nuclease-treated rabbit reticulocyte lysate was obtained from Promega (#L4960). Protease inhibitor cocktail (Cat. # PIC002) and AEBSF (Cat. # AEB602) were obtained from Bioshop Canada. E-64 (Cat. # E3132), bestatin hydrochloride (Cat. # B8385), leupeptin (Cat. # L2884), CA-074 (Cat. #205530), and aprotinin (Cat. # A1153) were obtained from Millipore-Sigma. Z-VAD-FMK (Cat. # ALX-260-020-M001), Q-VD-OPh (Cat. # S7311), acetyl-calpastatin 184–210 (Cat. # 2950), MDL-28170 (Cat. # S7394), calpeptin (Cat. # 0448), LY3000328 (Cat. # 29729), SB 412515 (Cat. # 23249), L-006,235 (Cat. # 28843), recombinant human cathepsin B (Cat.# 28843), and recombinant human cathepsin L (Cat.# 787504) were obtained from Cedarlane Laboratories. Mouse polyclonal anti-GFP epitope tag (Cat. # MMS-118R) was obtained from Covance, and rabbit polyclonal anti-GFP (Cat. #290) was obtained from Abcam.

### Purification of HIFαODD-GFP

*E. coli* BL21 (DE3) cells expressing pET21b-HIF1αODD-cp8GFP-35-His_6_, pET21b-HIF1αODD-cp8GFP-35-His_6_-M561T, pET21b-HIF1αODD-cp8GFP-35-His_6_-P402A/P564G, or pET21b-HIF2αODD-cp8GFP-35-His_6_ were grown in 1L LB containing 100 μg/mL ampicillin to an OD_600_ between 0.6 and 0.9. Protein expression was induced with 0.5mM IPTG and cells were grown for an additional 3h at 37°C before harvesting by centrifugation. Cell pellets were resuspended in lysis buffer (50 mM Tris–HCl, 150 mM NaCl pH 7.5, 5 mM imidazole) containing 2 mM β-mercaptoethanol and 1X protease inhibitor cocktail and homogenized by passing three times through an Emulsiflex-C3 cell disruptor (Avestin) at 20–30 kPSI. Lysates were clarified by centrifugation at 30,000 × g, 45 min and supernatants were applied to a 2 mL Ni–NTA (Thermo Scientific) column pre-equilibrated with lysis buffer. Beads were washed twice with 1X TBS (50mM Tris–HCl, 150 mM NaCl pH 7.5) containing 40 mM imidazole and 2 mM β-mercaptoethanol, then eluted with 6 mL of 1X TBS containing 0.5 M imidazole and 2 mM β-mercaptoethanol. Eluates were concentrated using an Amicon® Ultra-4 centrifugal filter with a 30 kDa cutoff (Millipore), and the concentrated protein was purified using a Superdex 200 Increase 10/300 GL (Cytiva).

### Cleavage of HIFαODD-GFP

Cleavage reactions were performed using 1.5 μL rabbit reticulocyte lysate and 1 μg HIF1αODD-GFP, HIF1αODD-GFP-M561T, HIF1αODD-GFP-P402A/P564G, or HIF2αODD-GFP in a 20 μL reaction. Protease inhibitor cocktail was used at the manufacturer’s recommended concentration (1X), and individual protease inhibitors (AEBSF, bestatin, E-64, leupeptin, aprotinin) were added at the concentrations present in the protease inhibitor cocktail. For caspase, calpain, and cathepsin inhibitor experiments, rabbit reticulocyte lysate was pre-treated with inhibitor or DMSO vehicle control for 5 min at 37°C, then added to 1 μg HIF1αODD-GFP in a 20 μL reaction. Final DMSO concentration was 0.1%. For pH dependence experiment, HIF1αODD-GFP was incubated with rabbit reticulocyte lysate in a 20 μL reaction containing either 50 mM sodium acetate/0.1% acetic acid pH 5; 50 mM MES pH 6; 50 mM HEPES pH 7; or 50 mM Tris–HCl pH 8. Reactions were incubated for 60 min at 37 °C.

For cleavage reactions using recombinant human cathepsins B and L, 1 μg HIF1αODD-GFP or HIF1αODD-GFP-M561T was diluted in 50mM MES pH 6 containing 5 mM DTT. Cathepsin B had an advertised specific activity of > 2,500 pmol/min/µg towards substrate Z-Leu-Arg-AMC and was added to 20 μL reactions in increasing amounts (0.16, 0.8, 4, 20 ng). To maintain comparable amounts of cathepsin L (advertised specific activity of > 23,000 pmol/min/µg towards Z-Leu-Arg-AMC), tenfold lower amounts of cathepsin L were added (0.016, 0.08, 0.4, 2 ng). Reactions were incubated for 1h at 37 °C.

### Western blotting

Reactions were terminated by the addition of 3X sample buffer; samples were heated to 100°C for 5 min, then were resolved on SDS-PAGE under reducing conditions. Proteins were transferred to PVDF membrane in 20% methanol, 22mM Tris, 171mM glycine and 0.01% SDS. Membranes were blocked for 1h at room temperature in TBS-T (20mM Tris pH 7.6, 137 mM NaCl, 0.05% Tween 20) containing 5% skim milk, and incubated overnight in primary antibody (anti-GFP), diluted 1:25,000 in TBS-T containing 5% BSA and 0.02% sodium azide. Membranes were washed in TBS-T, then were incubated for 45 min in secondary antibody (goat anti-mouse-HRP or goat anti-rabbit-HRP), diluted 1:30,000 in blocking buffer. Following three washes in TBS-T, membranes were developed using ECL substrate (Haan & Bermann, 2006) and visualized on a ChemiDoc™ MP Imaging System using ImageLab™ software (Bio-Rad).

### Sequence alignment

HIF1α (amino acids 548–585) and HIF2α (amino acids 514–553) protein sequences were aligned using Clustal Omega^66,67^.

### Supplementary Information

Supplementary Information.

## Data Availability

All data generated or analysed during this study are included in this published article [and its supplementary information file].
