# Supplementary material for: Cathepsins L and B target HIF1α for oxygen-independent proteolytic cleavage
Source: Sci Rep. 2024 Jun 26;14:14799. doi: 10.1038/s41598-024-65537-9 (PMC11208597; doi:10.1038/s41598-024-65537-9)

SUPPLEMENTARY INFORMATION

SUPPLEMENTARY FIGURE LEGENDS

Supplementary Figure S1. Rabbit reticulocyte lysate retains activity towards HIF1αODD across a wide pH range. HIF1αODD-GFP was incubated for 60 minutes at 37°C in the absence or presence of rabbit reticulocyte lysate (RRL) in buffers at the indicated pH. Reactions were resolved on SDS-PAGE and immunoblotted for GFP. Arrow indicates cleaved HIF1αODD-GFP. Asterisks indicate non-specific protein bands. Blot is representative of three independent experiments. Original blot is presented in Supplementary Fig. S2.

Supplementary Figure S2. Complete images of all immunoblots. Boxes denote where the original images were cropped.

Supplementary Figure S1

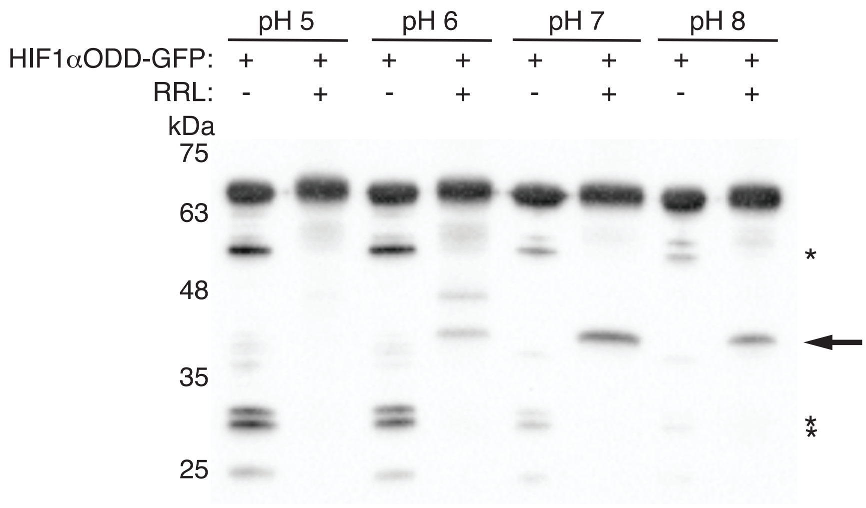

IB: anti-GFP

Supplementary Figure S2

Figure 1a

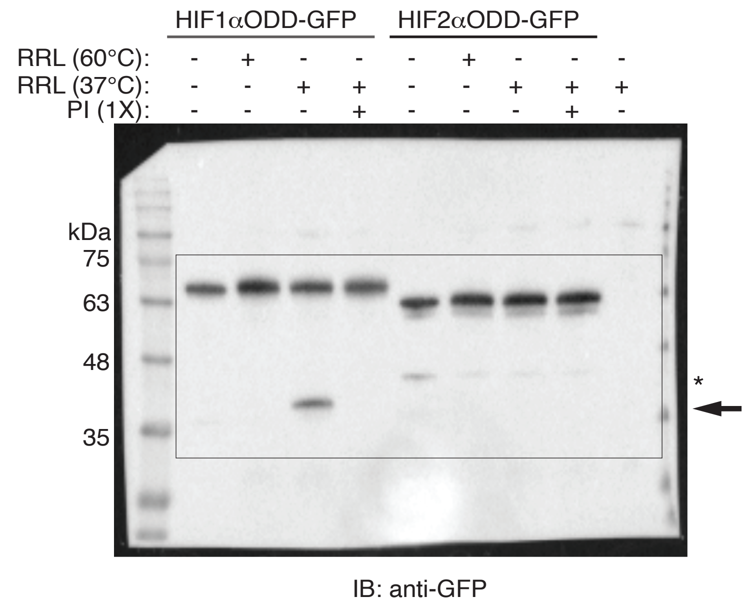

Figure 1b

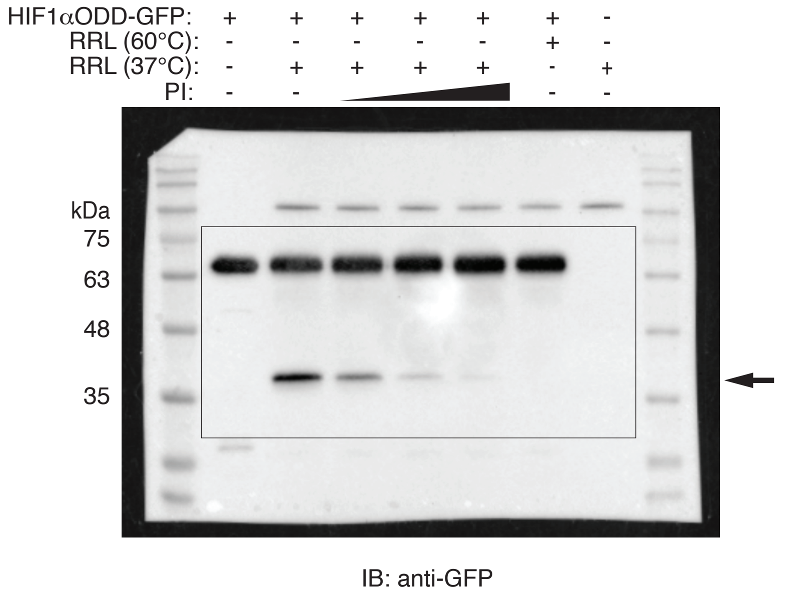

Figure 2a

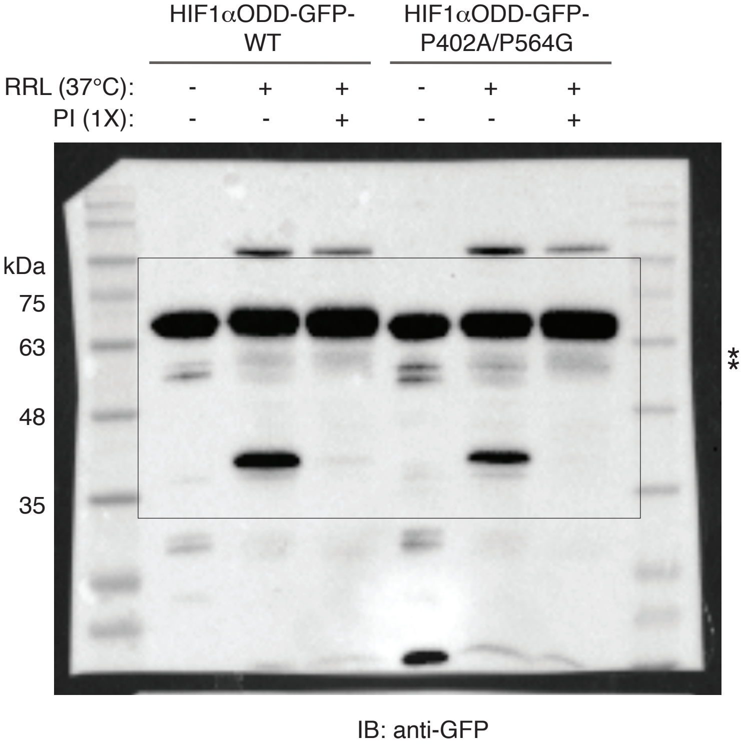

Figure 2c

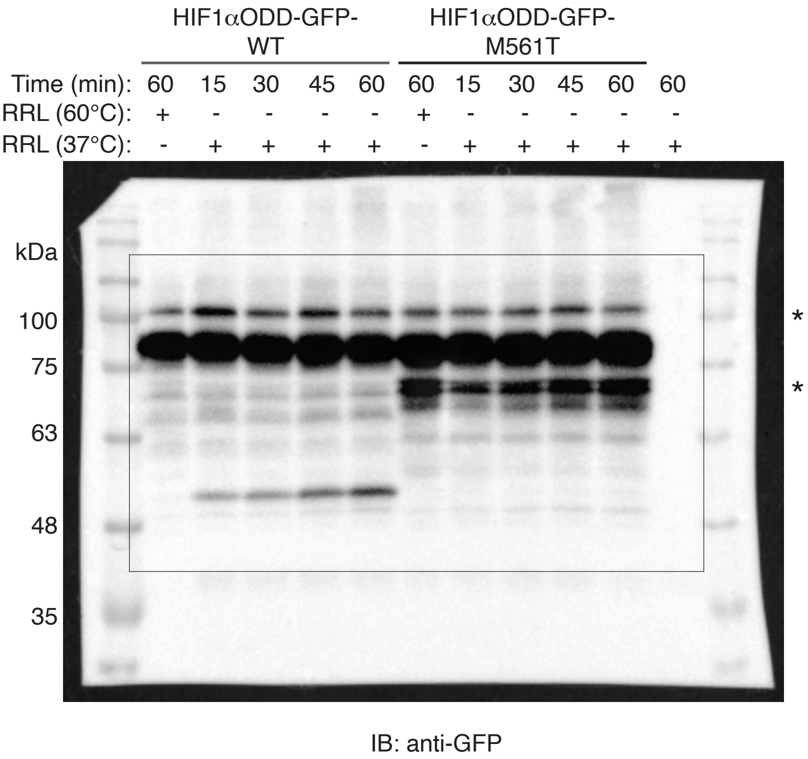

Figure 3a

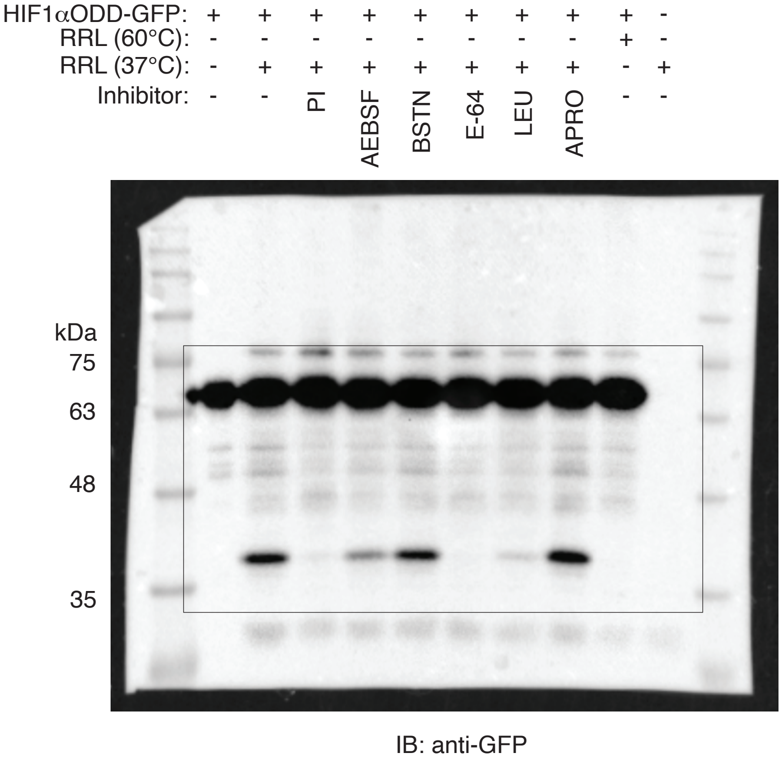

Figure 4a

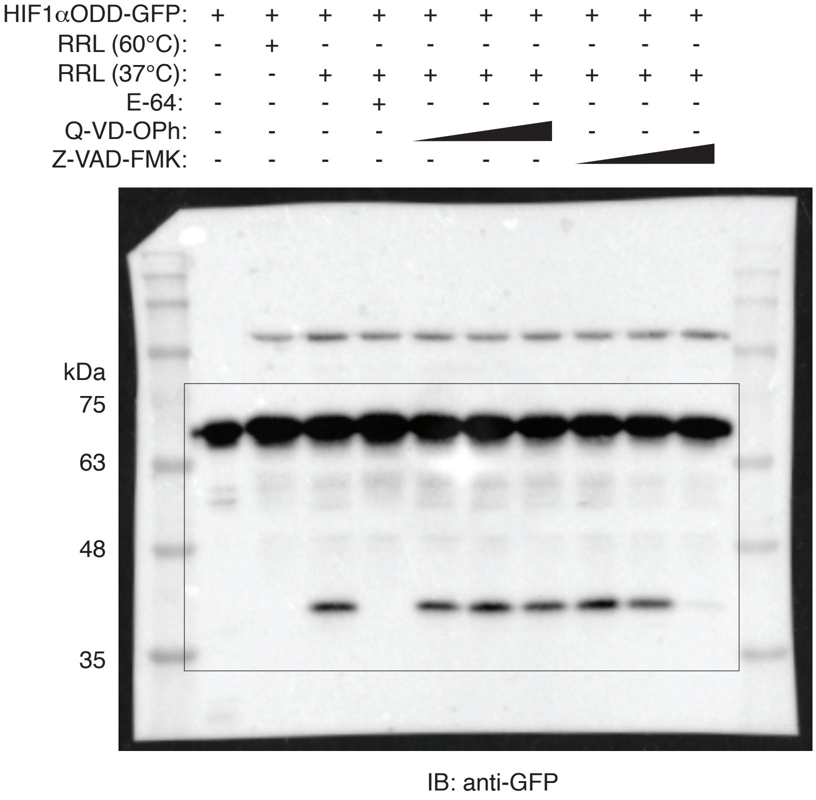

Figure 4b

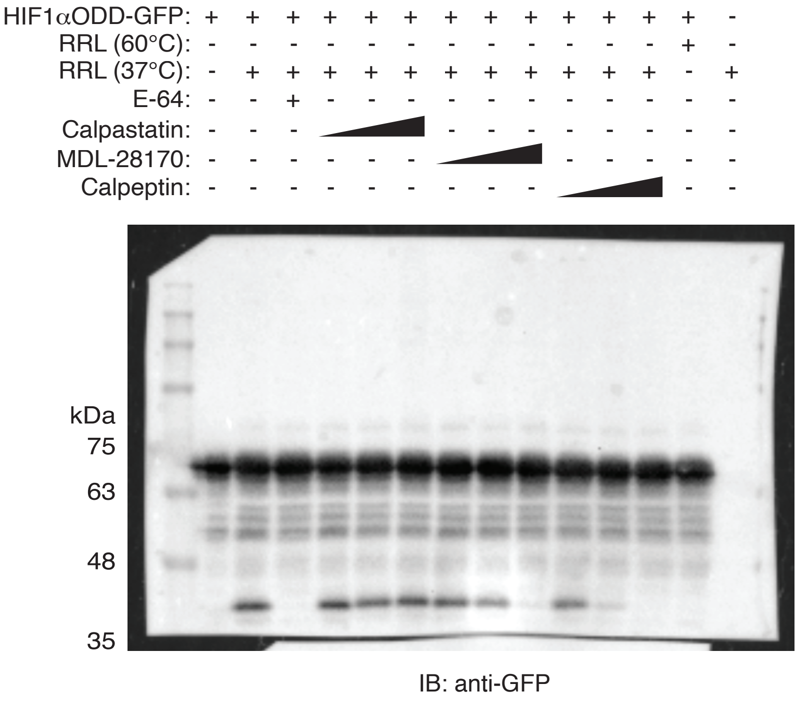

Figure 4c

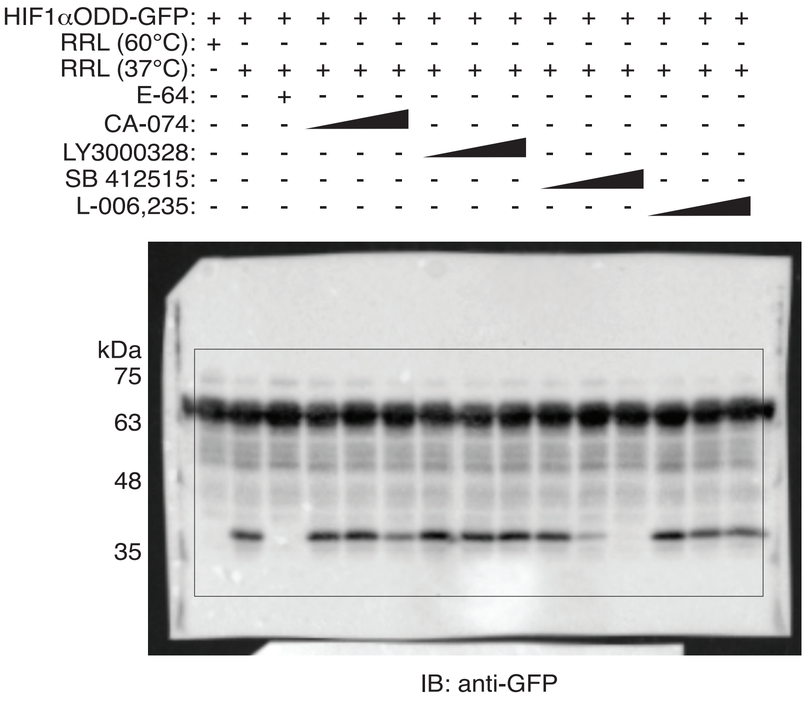

Figure 5a

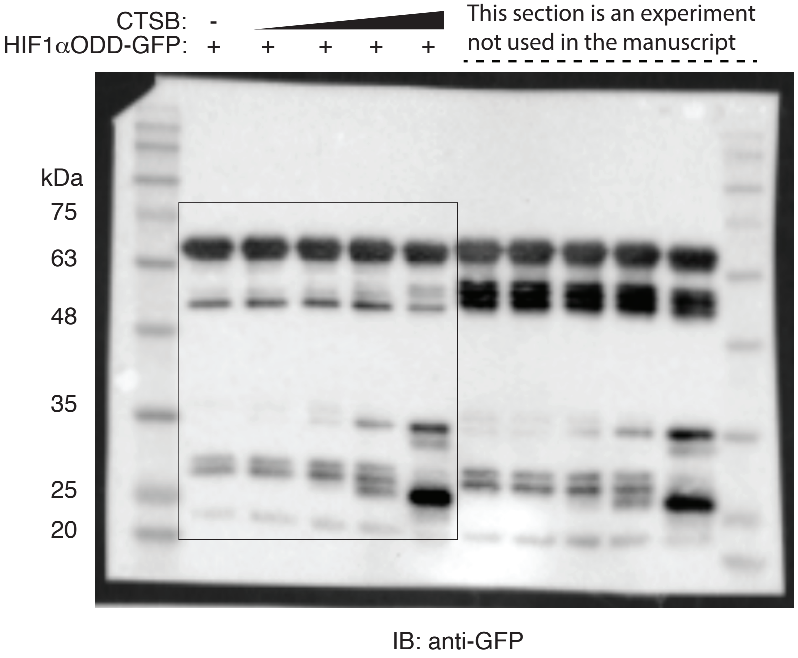

Figure 5b

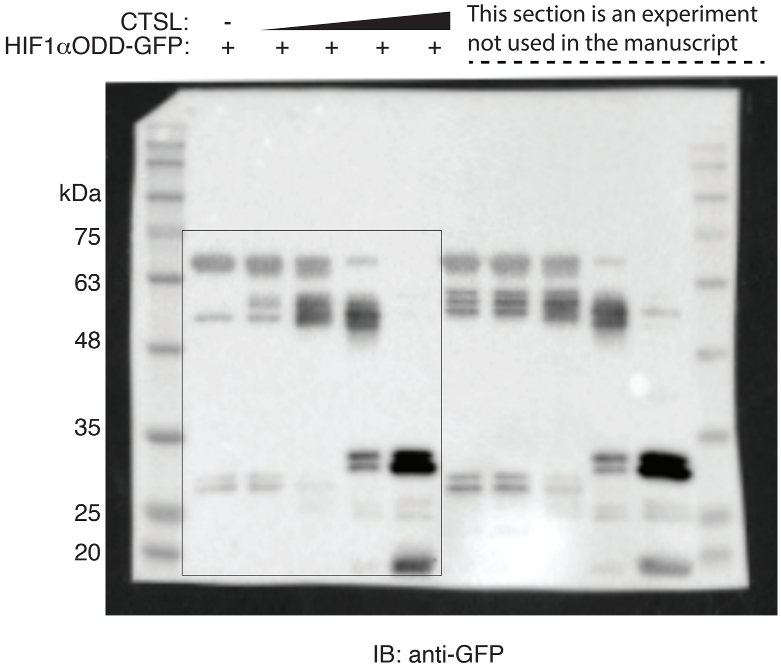

Supplementary Figure S1

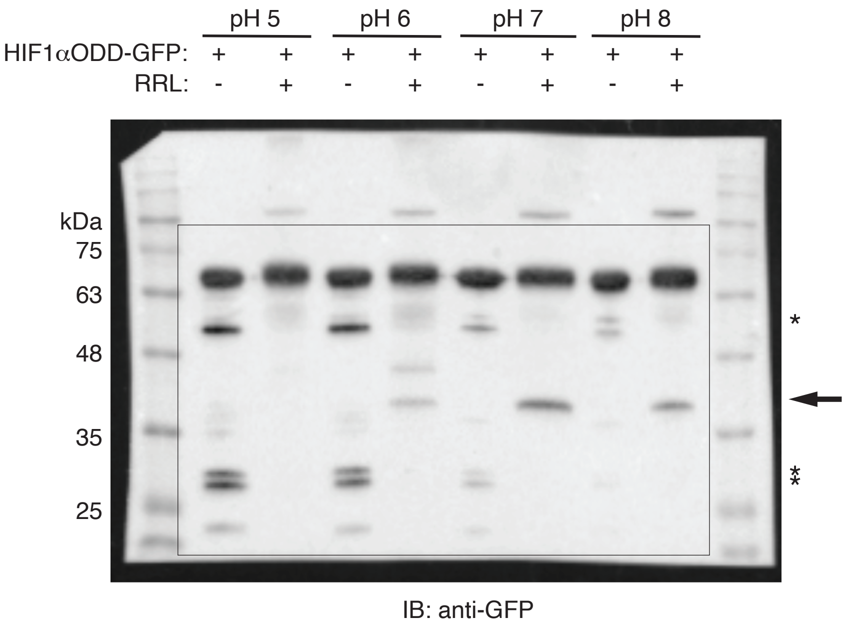

Supplement: Supplementary file 1 — Supplementary Information. [file 41598_2024_65537_MOESM1_ESM.pdf]
